# Factors influencing the habitat use by ocelots in one of the last large Atlantic Forest remnants in southeastern Brazil

**DOI:** 10.1002/ece3.7363

**Published:** 2021-03-13

**Authors:** Juliana Benck Pasa, Ricardo Corassa Arrais, Rodrigo Lima Massara, Gabriel Pereira, Fernando Cesar Cascelli de Azevedo

**Affiliations:** ^1^ Programa de Pós‐Graduação em Ecologia Universidade Federal de São João del Rei São João del Rei Brazil; ^2^ Programa de Pós‐Graduação em Ecologia Conservação e Manejo de Vida Silvestre Instituto de Ciências Biológicas Universidade Federal de Minas Gerais Belo Horizonte Brazil; ^3^ Laboratório de Ecologia e Conservação Departamento de Biologia Geral Universidade Federal de Minas Gerais Belo Horizonte Brazil; ^4^ Instituto SerraDiCal de Pesquisa e Conservação Belo Horizonte Brazil; ^5^ Departamento de Geociências Universidade Federal de São João del Rei São João del Rei Brazil; ^6^ Programa de Pós‐Graduação em Geografia Física Universidade de São Paulo Brazil; ^7^ Departamento de Ciências Naturais Universidade Federal de São João del Rei São João del Rei Brazil; ^8^ Instituto Pró‐Carnívoros Atibaia Brazil

**Keywords:** biodiversity hotspot, dry season, landscape features, mesocarnivore, native grassland areas, tropical rainforest

## Abstract

Ocelots (*Leopardus pardalis*) are widely distributed throughout the Americas, being dependent on forested areas to survive. Although ocelot ecology is broadly studied throughout the species range distribution, studies concerning factors that may affect ocelot occupancy in the Atlantic Forest are still scarce. We used camera traps to evaluate factors influencing the probabilities of detection and occupancy of ocelots in a protected area of the Atlantic Forest, the Rio Doce State Park (RDSP), southeastern Brazil. To assess ocelot occupancy and detection probabilities, we measured the distances between sampling stations and rivers, lakes, cities, pasture, and Eucalyptus plantations. In addition, we recorded the mean rainfall levels for each sampling occasion, and native grassland areas within a 500 m‐buffer around each sampling station. We found a strong and positive association between ocelot detection and the dry season, which might be due to a higher number of individuals moving through the Park during this season. Moreover, we found a strong and positive association of ocelot detection with native grassland areas around lakes, which may be related to the ocelot behavior of searching for prey in these areas. Conversely, the ocelot occupancy probability was intermediate ($\hat{\Psi }$ = 0.53, 95% CI = 0.36–0.69) and was not strongly associated with the evaluated covariates, which may be explained by the high‐quality of forest habitats and water resources that are homogeneously distributed within the Park. Our study indicates that the RDSP still provides a structurally suitable forest habitat for ocelots, but because of the current worrying scenario of over fragmentation, reduction of forest cover, and weakness of the protective legislation of this biome, the long‐term persistence of the species in RDSP is uncertain.

## Graphical abstract

We used camera traps to evaluate factors influencing the probabilities of detection and occupancy of ocelots in a protected area of the Atlantic Forest, the Rio Doce State Park (RDSP), southeastern Brazil. We found a strong and positive association between ocelot detection and the dry season and native grassland areas around lakes, and these associations may be related to a higher number of individuals moving through the Park during this season and a behavior of searching for prey in native grassland areas. Conversely, the ocelot occupancy probability was intermediate (Ψ ^ = 0.53, 95% CI = 0.36–0.69) but not strongly associated with the evaluated covariates, which may be explained by the high‐quality of forest habitats and water resources that are homogeneously distributed within the Park. Our study indicates that the RDSP still provides a structurally suitable forest habitat for ocelots, but because of the current worrying scenario of over fragmentation, reduction of forest cover and weakness of the protective legislation of this biome, the long‐term persistence of the species in RDSP is uncertain.

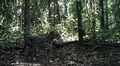

## 1 INTRODUCTION

In recent decades, numerous ecosystems have suffered a severe loss and fragmentation, culminating in a population decline of wild mammalian carnivore species worldwide (Karanth & Chellam, 2009; Loveridge et al., 2010). In general, mammalian carnivores occur in low density, since they need large areas to survive (Karanth & Chellam, 2009; Loveridge et al., 2010). Their populations decline may lead to several ecosystem imbalances, considering that these species are efficient prey regulators (Azevedo & Verdade, 2012; Terborgh et al., 2001). In current reduced and fragmented natural areas, some species of more resilient mammalian carnivores use areas modified by humans or in a regeneration stage to prey searching (Karanth & Chellam, 2009). However, in general, the occurrence of mammalian carnivores in these landscapes is often restricted to the presence of food, water resources and the remaining natural vegetation (Boron et al., 2018; Cruz et al., 2019; Gompper et al., 2016; Massara et al., 2018).

The Atlantic Forest is originally one of the largest tropical forests in the Americas, a biodiversity hotspot sustaining a high biological diversity (Laurance, 2009; Silva & Casteleti, 2003). However, due to human expansion, the Atlantic Forest is in a process of loss and fragmentation, in which only 12.4% of the original coverage remains, with the majority (>80%) of the remnants having less than 50 hectares (Ribeiro et al., 2009; SOS Mata Atlântica, 2019). In this scenario, larger and more connected Atlantic Forest remnants are important for maintaining biodiversity (Ahumada et al., 2011; Magioli et al., 2015; Ribeiro et al., 2009). In the state of Minas Gerais, southeastern Brazil, the largest remnant of the Atlantic Forest is the Rio Doce State Park (RDSP) (IEF, 2019). RDSP is important in the maintenance of several ecosystem services, by protecting a large area of continuous high‐quality forest, abundant water bodies, and a great diversity of fauna (IEF, 2019).

The ocelot (*Leopardus pardalis*) is the third largest felid in Latin America (Sunquist & Sunquist, 2002), presents a solitary and elusive behavior, and an opportunistic diet (Azevedo et al., 2019; Silva‐Pereira et al., 2011). Ocelots are widely distributed across the Americas, occurring from southern Texas in the United States to northern Argentina (Murray & Gardner, 1997). In Brazil, ocelots occur in almost the entire country (Murray & Gardner, 1997). Ocelots are dependent on habitats with high vegetation cover, preferring protected areas composed of extensive forests (Di Bitetti et al., 2010; Emmons, 1998; Massara et al., 2015; Sunquist & Sunquist, 2002). In this context, ocelots can avoid open areas (e.g., grassland) of low vegetation cover (Boron et al., 2018; Cruz et al., 2019), while their occurrence can be favored in sites with dense canopy and understory coverage (Haines et al., 2006; Paolino et al., 2018; Wolff et al., 2019). In addition, ocelots can be strongly associated with the proximity of rivers and lakes (Wang et al., 2019; Wolff et al., 2019) and may also respond to variation in climate parameters. In terms of climate effects, the variation in rainfall levels can influence the availability of ocelot prey species, since during the rainy season this availability may be greater (Dillon & Kelly, 2008; Sunquist & Sunquist, 2002), causing the species to move less frequently compared to the dry season. Anthropogenic effects may also affect ocelots' occurrence. For instance, proximity to cities, Eucalyptus plantations and pasture can increase contact between humans and wildlife, negatively influencing the occurrence of ocelots and putting their survival and long‐term persistence in these forest remnants at risk (Dotta & Verdade, 2011; Loveridge et al., 2010; Wang et al., 2019).

Although an extensive knowledge about ocelot´s ecology could be found in the literature (Azevedo et al., 2019; Bianchi et al., 2012; Di Bitetti et al., 2006, 2008; Goulart, Graipel, et al., 2009; Massara et al., 2015, 2016; Santos et al., 2014), few studies have addressed the habitat use by the species, particularly in protected areas of the Atlantic Forest (Di Bitetti et al., ,,^2006^, ^2010^; Goulart, Cáceres, et al., 2009; Massara et al., 2018). In a recent study conducted in six protected Atlantic Forest reserves (including RDSP) addressing ocelot´s occupancy (Massara et al., 2018), the adopted sample design was restricted only to some portions of the study areas, thus limiting the understanding of the impact of some environmental variables on ocelot´s occupancy and detection in each area separately.

We performed a study of ocelots by sampling the entire area of RDSP to evaluate the influence of habitat features on the detection and occupancy probabilities of the species. Detection probability may be influenced by methodological factors, such as sampling effort, but also vary spatially due to habitat characteristics and temporarily due to seasonal fluctuations (e.g., food resource) that may affect the movement of the species (Bailey et al., 2004; Gu & Swihart, 2004). Thus, here we interpreted detection probability as the frequency (or intensity) of use of sampling stations by ocelots (Dias et al., 2019a, 2019b; Massara et al., 2018). Specifically, we hypothesized that both detection and occupancy probabilities would be (a) negatively influenced by native grassland (some grassland areas around lakes inside the Park) and (b) positively influenced by either a closer proximity to rivers and lakes or by a higher distance to cities pasture and Eucalyptus plantations. Additionally, we evaluated the influence of the climate on the ocelot detection probability and hypothesized that detection probability would be (c) negatively influenced by higher levels of rainfall.

## 2 MATERIALS AND METHODS

### 2.1 Study area

**FIGURE 1:**
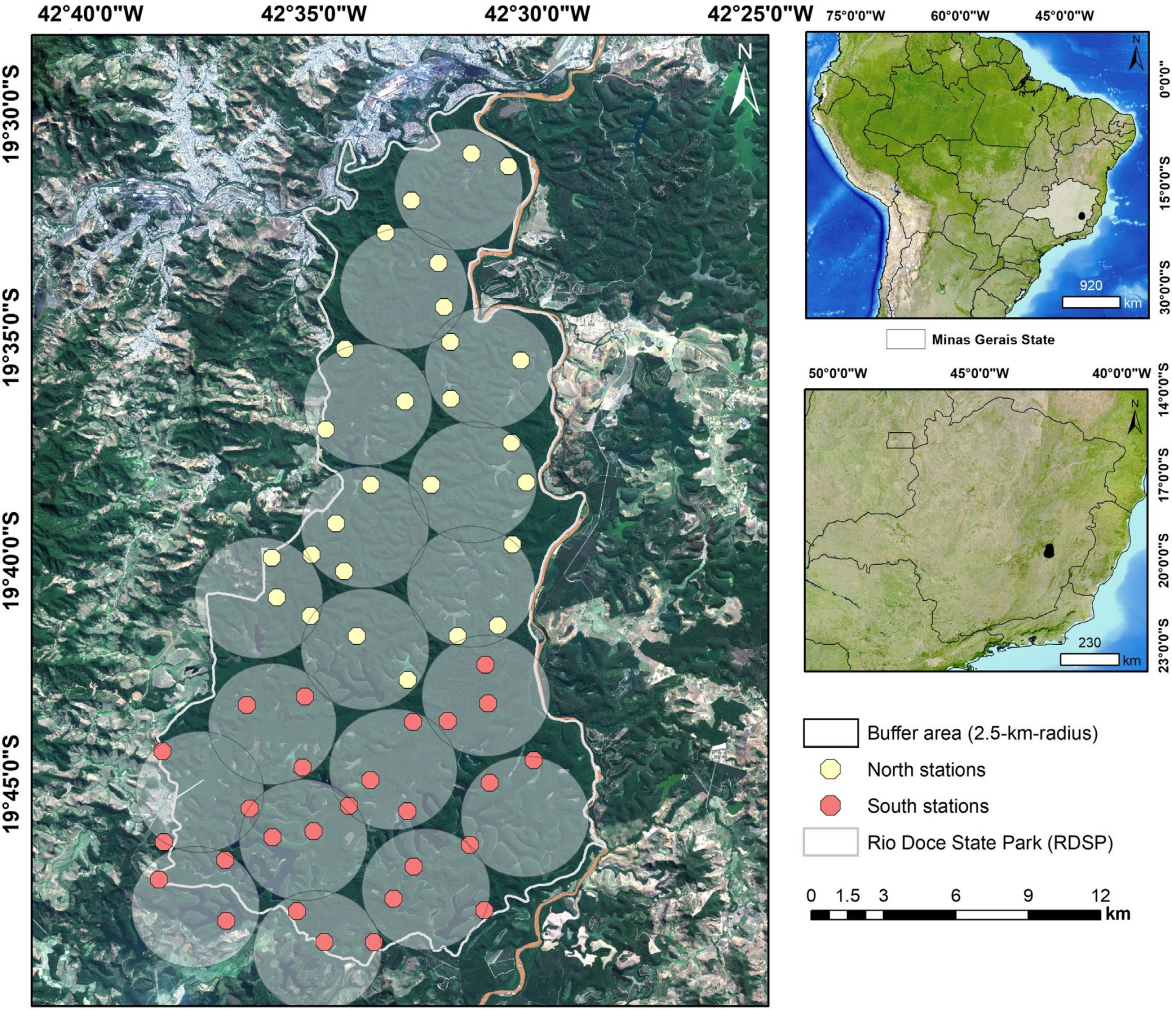
Distribution of buffers and stations in Rio Doce State Park during the ocelot camera trapping study. Yellow and red circles represent camera stations installed during random placement in north and south sectors, respectively. Inserts show the position of the state of Minas Gerais in Brazil and the position of the Rio Doce State Park. Geographic coordinate system: SIRGAS 2000 UTM_Zone_23S. Source: IBGE 2018

The study was conducted in RDSP, state of Minas Gerais, southeastern Brazil (Figure 1). The Park covers approximately 360 km^2^, representing one of the largest continuous remnants of Atlantic Forest in Brazil and the largest in the state of Minas Gerais (Gontijo & Britto, 1997). In addition to ocelots, the RDSP includes a variety of medium and large‐sized mammal species, such as jaguars (*Panthera onca*), pumas (*Puma concolor*), margay cats (*Leopardus wiedii*), tapirs (*Tapirus terrestris*), and giant armadillos (*Priodontes maximus*) (Keesen et al., 2016; Stallings et al., 1991). The RDSP has 42 natural lakes located mainly in the southern portion of the Park, three streams (Belém in the north, Turvo in the middle, and Mombaça in the south), and rivers Piracicaba and Doce bordering some areas of the Park. The RDSP represents an important area for maintenance of biodiversity in the Atlantic Forest (Silva Júnior et al., 2010), and the vegetation is classified as submontane seasonal semideciduous forest (Lino & Dias, 2014). The climate is classified as humid subtropical (IBGE, 2002), with dry (April–September) and rainy periods (October–March) (Pereira et al., 2018). Human‐altered environments around the Park are composed mainly of Eucalyptus plantations, pasture, and urban areas (PELD/CNPq, 2007).

### 2.2 Sampling design

Originally, our sampling design was set to apply capture‐recapture models for estimating abundance and density of jaguars for the RDSP. Thus, we divided the study area into two sectors: north and south, each including nine circular zones (buffers) that were 5.0‐km in diameter covering the entire RDSP area. This size represents twice the size of the smallest conservatively estimated home range size for female jaguars (i.e., 10‐km^2^) in a Central America tropical forest habitat (Rabinowitz & Nottingham, 1986). In each buffer, we used a random number generator to define three random locations, indicating where the camera traps should be installed (Figure 1), resulting in 27 stations per sector and a total of 54 stations in both sectors. We used a minimum distance of 1.5‐km between stations. Every time a selected point fell less than 1.5‐km from another one inside a buffer, it was discarded and another point was randomly generated, leading to a suitably spaced camera distribution and implying that our random sampling design was restricted to ensure adequate spatial coverage. No sampling station was located along roads or trails. We used camera traps (Bushnell© Trophy Cam Natureview, Trophy Cam Standard, and Trophy Cam Essential ‐ Kansas, USA) to establish capture stations (hereafter “stations”). Stations included a pair of camera traps installed at 40–50 cm in height that were fixed to trees and facing each other. Cameras were set to record 10–30 s HD videos, with an interval of 60 s between videos. All cameras were set to operate simultaneously for 24 hr/day, over a period of 40 days in each season (dry and rainy), totaling 80 days of camera data. We did not use baits or any kind of attraction. Due to a lack of roads and access in remote areas of the RDSP, we covered 340 km of man‐made trails to access the designated stations.

**FIGURE A1:**
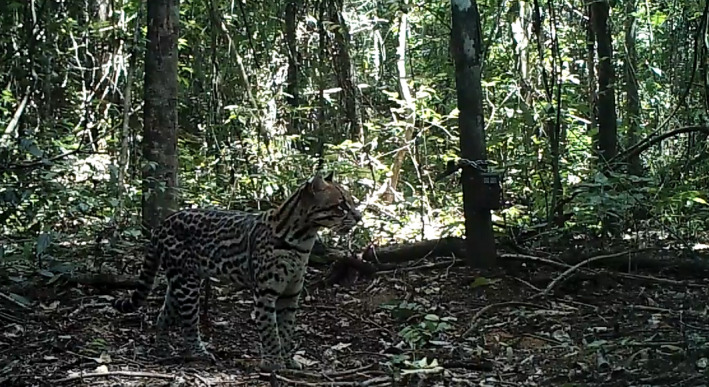
Ocelot detected by a camera trap during the dry season, in a station located at the southern portion of Rio Doce State Park, state of Minas Gerais, Brazil

The same stations were sampled in both dry and rainy seasons. In each season, stations were installed for 40 days in the northern RDSP sector, followed by removal and installation in the southern sector for more 40 days, within a maximum of 120 days of sampling for both sectors in each season (Figure A1 in Appendix), totally approximately 240 days of sampling. The entire survey (dry and rainy) occurred over 11 months. The setup/takedown periods lasted ~30 days, allowing the period for active data collection from cameras to span the dry (30 April–25 August 2016) and rainy (25 November 2016–7 March 2017) seasons. We only considered camera trap data during the period when all cameras were functional at the same time for each season.

### 2.3 Habitat covariates and Landscape structure

For each station, we determined five station‐specific covariates: distance to the nearest river, distance to the nearest lake, distance to the nearest city, distance to the nearest pasture, and distance to the nearest Eucalyptus plantation (Table 1). Distances between the sampling stations and rivers, lakes, cities, pasture, and Eucalyptus plantations were measured in meters, using Sentinel‐2 satellite images (10 m spatial resolution) from 2016 in ArcGIS 10.5 (ESRI, 2016) and SPRING 5.3 (Camara et al., 1996).

**TABLE 1:** Covariates used to model the occupancy (Ψ) and detection (*p*) probabilities of ocelots in the Rio Doce State Park, Brazil, and their expected effects

Covariates	Parameter	Expected effect
Distance to the nearest river	Ψ*, p*	Higher occupancy and detection probabilities of ocelots closer to rivers and lakes. Ocelots might use water resources to meet their water requirements and also for prey searching (Di Bitetti et al., 2008; Nagy‐Reis et al., 2017; Sunquist & Sunquist, 2002)
Distance to the nearest lake	Ψ*, p*	
Distance to the nearest city	Ψ*, p*	Lower occupancy and detection probabilities of ocelots closer to cities, pasture and Eucalyptus plantations. These human‐altered habitats cause reduction and fragmentation of ocelots' native habitats, and also increase contact between humans and wildlife (Cruz et al., 2019; Dotta & Verdade, 2011; Loveridge et al., 2010)
Distance to the nearest pasture	Ψ*, p*	
Distance to the nearest Eucalyptus plantation	Ψ*, p*	
Native grassland areas	Ψ*, p*	Lower occupancy and detection probabilities of ocelots in native grassland areas. Ocelots prefer to use habitats with a denser vegetation cover (e.g., forests) to hunt, refuge and movement (Lyra‐Jorge et al., 2010; Paolino et al., 2018; Wang et al., 2019)
Mean rainfall	*p*	Lower detection of ocelots with higher levels of rainfall. Ocelots' prey species reproduce in rainier periods (Catzeflis et al., 2019), thus increasing prey availability and reducing ocelot's movements through the environment (Massara et al., 2015)

**FIGURE A2:**
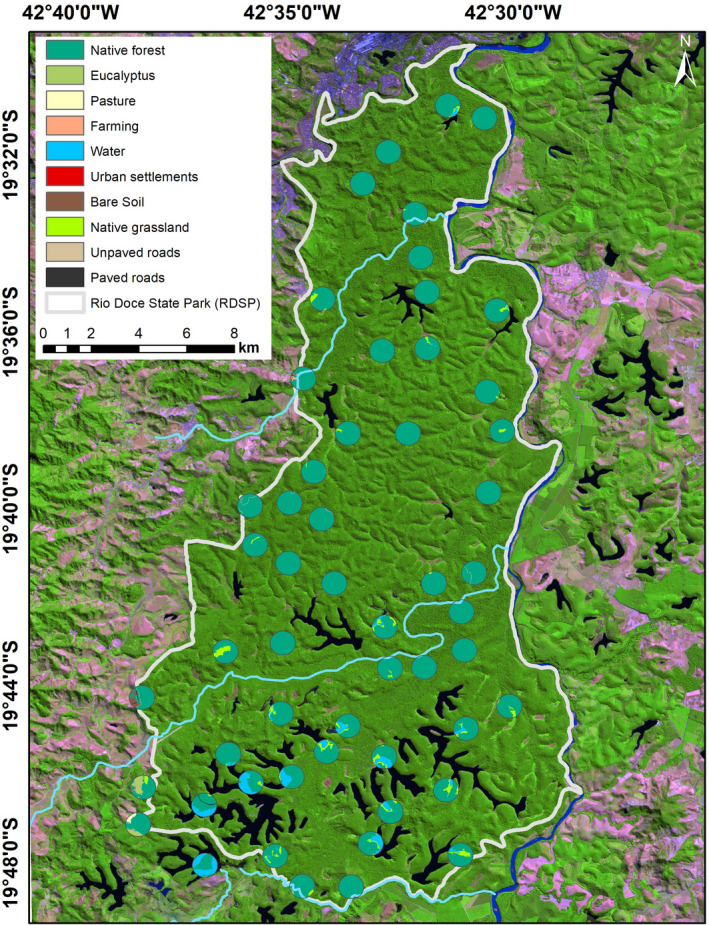
Land use types within the buffer areas (500‐m radius) surrounding each sampling station in Rio Doce State Park, state of Minas Gerais, southeastern Brazil. The categorization of the different land use types within the buffer zones was used to calculate the area (in hectares) from each land use type. The grassland areas were used to assess their influence on ocelot occupancy and detection probabilities in Rio Doce State Park

Likewise, to characterize the surrounding habitat of each station, we generated a land use and land cover (LULC) map for the region encompassing RDSP using a supervised classification of Sentinel‐2 satellite images (10 m spatial resolution) from 2016. We classified landscape cover into the following categories: forest, native grassland, houses, paved roads, Eucalyptus plantations, planted pasture, bare soil, and unpaved roads. Forested areas were characterized by secondary or primary forest, which are present in approximately 94% of RDSP (Oliveira et al., 2019). Grassland areas were characterized by native grass vegetation, which are present in places where the lakes have reduced in size or dried up completely and corresponded to 0.7% of RDSP. Eucalyptus plantations were located only outside RDSP. Our final map consisted of a raster file with 10‐m pixel sizes for all the landscape. Based on ground‐truthing, our map´s accuracy was >95%. For each station, we selected a concentric circle (buffer) of 500‐m radius (Figure A2 in Appendix) (Lombardi et al., 2020). This extent covers 78.5 ha and is equivalent to the smallest home range size ever recorded for ocelots (76 ha; Crawshaw & Quigley, 1989). Within the buffers, we estimated the total area of all habitat categories (Figure A2 in Appendix). However, all categories of habitats, except native grasslands, showed little variation between the stations, and thus, we excluded these covariates from our analyses (Figure A2 in Appendix).

We estimated the effect of mean rainfall on detection probabilities using data obtained from the National Meteorological Institute, recorded by the meteorological station of the municipality of Timóteo (INMET, 2018), located about 6 km from RDSP. We collected daily rainfall values (mm) and generated a mean value of rainfall for each sampling occasion of each station. We used these values as indexes of mean precipitation over the entire RDSP.

### 2.4 Data analyses

We combined detections into 5‐day periods (sampling occasions) to build the detection history for each station, through recording whether the species was detected (1) or not (0) by either camera. Using this data, we first evaluated changes in occupancy state between seasons (i.e., evaluated the closure assumption) using a dynamic occupancy model approach (MacKenzie et al., 2003). This model approach allowed us to evaluate whether or not a model that estimates the parameters of extinction (epsilon) and local colonization (gamma) of the stations by ocelots between seasons fit better than a model where these parameters were fixed to zero (Rota et al., 2009). Specifically, we fit two models, where the parameters of local colonization and extinction were either estimated (alternative hypothesis; open population) or fixed to zero (closed population or null hypothesis; that is, occupancy state of the stations is static between seasons) (e.g., Massara et al., 2018; Nagy‐Reis et al., 2017). Using the Akaike Information Criterion adjusted for small sample sizes (AICc) (Burnham & Anderson, 2002), the best‐supported model was the open population (ΔAICc = 6.5 for the next best model, which included colonization and extinction fixed at 0), revealing a change in the state of occupancy between seasons. Because our objective was not to evaluate the population dynamics of ocelots between seasons and we had a limited temporal sample size (*n* = 2 seasons), we included the categorical covariate season (stations sampled during dry = 0; and rainy = 1) in the set of variables to account for changes in the state of occupancy between seasons, and also included this covariate to account for changes in the detection probabilities. Thus, we used a single‐season occupancy model for subsequent analysis (Mackenzie et al., 2002). We separated dry and rainy sampling occasions, yielding a total of 8 sampling occasions for each station and for each season. Our models consisted of two parameters: the occupancy probability (Ψ), which is defined as the probability of a sampling station *i* is occupied by ocelots; and the detection probability (*p*), which is defined as the probability of detecting ocelots at the sampling station *i* at time (or sampling occasion) *t*, given it is occupied (Mackenzie et al., 2002).

We evaluated for a possible lack of independence (i.e., overdispersion) between sampling stations, performing the overdispersion test (MacKenzie & Bailey, 2004) available in Program PRESENCE 2.12.36 (Hines, 2006) using the model that contained the largest number of covariates (i.e., global or most parameterized model; MacKenzie & Bailey, 2004). No violation of the premise of independence between sampling stations was revealed (*χ* ^2^ = 282.48; *p* = .21; *ĉ* = 1.16). To investigate which covariate influenced the probabilities of occupancy (Ψ) and detection (*p*) of ocelots (Table 1), all possible additive combinations of models were constructed (Doherty et al., 2012). We limited the models to have 4 covariates or less; thus, the models had a maximum of 6 estimated beta parameters, resulting in a final set of 1,941 models. This model construction allowed us to obtain a balanced model set to interpret the cumulative AICc weights (*w*_+_) for each covariate. We considered covariates with *w*_+_ ≥ 0.50 as having strong influence on occupancy and detection probabilities (Barbieri & Berger, 2004). We built the models in Program MARK (White & Burnham, 1999) and ranked candidate models using the AICc (Burnham & Anderson, 2002). When different models were equally plausible (ΔAICc ≤ 2), our final average estimates for the occupancy and detection parameters were based on the model‐averaged estimates and the maximum likelihood methods incorporated into program MARK (Burnham & Anderson, 2002; Mackenzie et al., 2018). We evaluated the correlation among covariates using the Pearson correlation test to exclude highly correlated covariates (*r* ≥ 0.6) (Wang et al., 2019). Because no covariates were highly correlated, we kept them in the analysis (Table A1 in Appendix).

## 3 RESULTS

We detected ocelots in 23 sampling stations during the dry season (naïve occupancy = 0.43) and in 15 sampling stations during the rainy season (naïve occupancy = 0.28). Stations were in average 5,856.2 m (range = 517.9–14,929.9 m) distant to the nearest river, 1,292.4 m (0.00–3,977.2 m) to the nearest lake, 5,854.38 m to the nearest city (347.26–11,116.16 m), 3,414.49 m to the nearest pasture (684.11–4,701.97 m), and 2,918.63 m to the nearest Eucalyptus plantation (127.28–6,585.39 m). Mean rainfall levels were 0.20 mm (*SE* = 0.02) for the dry and 6.08 mm (*SE* = 0.38) for the rainy season. 

**TABLE 2:** Model selection results for the top 15 models composed of the occupancy (Ψ) and detection (*p*) probabilities of ocelots in the Rio Doce State Park, southeastern Brazil

Model	AICc	ΔAICc	AICc weights	Number of parameters	Deviance
Ψ(.), *p*(season + grass)	436.76	0.00	0.04	4	428.38
Ψ(past), *p*(season + grass)	437.59	0.82	0.02	5	427.00
Ψ(.), *p*(season + rain + grass)	437.97	1.21	0.02	5	427.38
Ψ(.), *p*(season + river + grass)	438.04	1.28	0.02	5	427.45
Ψ(.), *p*(season + lake + grass)	438.23	1.47	0.02	5	427.64
Ψ(.), *p*(season + grass + euc)	438.30	1.54	0.02	5	427.71
Ψ(.), *p*(season + grass + past)	438.64	1.87	0.01	5	428.05
Ψ(grass), *p*(season + grass)	438.64	1.87	0.01	5	428.05
Ψ(lake), *p*(season + grass)	438.68	1.91	0.01	5	428.09
Ψ(past), *p*(season + rain + grass)	438.79	2.02	0.01	6	425.95
Ψ(past), *p*(season + lake + grass)	438.82	2.05	0.01	6	425.99
Ψ(river), *p*(season + grass)	438.84	2.07	0.01	5	428.25
Ψ(euc), *p*(season + grass)	438.95	2.18	0.01	5	428.36
Ψ(city), *p*(season + grass)	438.95	2.19	0.01	5	428.37
Ψ(season), *p*(season + grass)	438.95	2.19	0.01	5	428.37

Note

The models were selected using the Akaike Information Criterion adjusted for small samples (AICc). The occupancy and detection probabilities were modeled according to the season; native grassland areas (grass); distances between the sampling station and the nearest river (river), the nearest lake (lake), the nearest pasture (past), the nearest Eucalyptus plantation (euc), and the nearest city (city). In addition, the detection probability only was also modeled according to the mean rainfall (rain) in each sampling occasion. The signal "+" means an additive effect between more than one evaluated covariate, and signal "." means absence of covariates (i.e., only the intercept).

**FIGURE 2:**
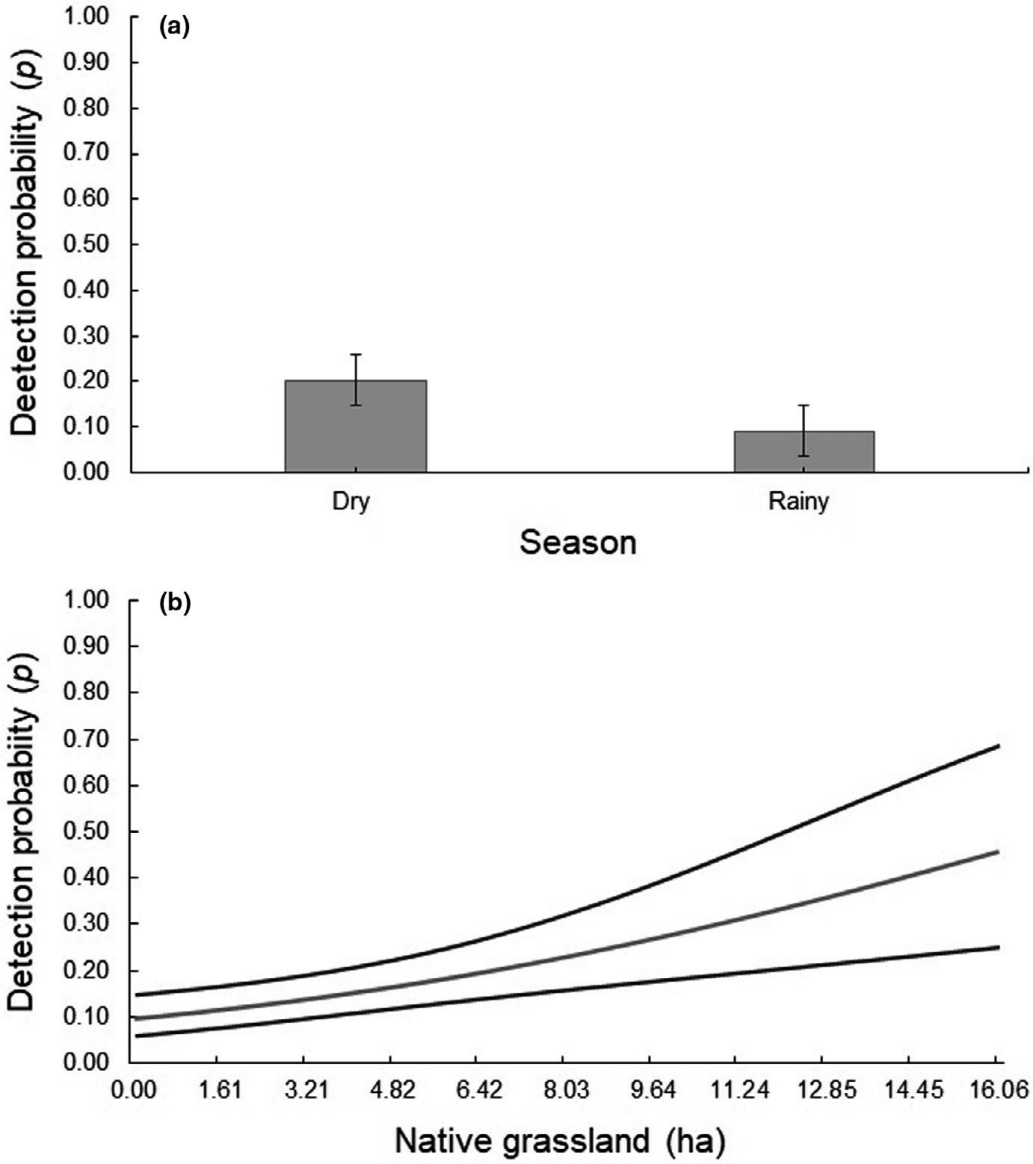
Ocelot detection probabilities (±95% CI) in the Rio Doce State Park, state of Minas Gerais, southeastern Brazil, in function of (a) season and (b) native grassland (in ha). The detection probabilities estimates were derived from the best ranked model containing the covariate

The model‐averaged estimates resulted in an occupancy probability of 0.53 (95% CI = 0.36–0.69) and in a detection probability of 0.13 (95% CI = 0.08–0.19; Table 2). No covariate influenced the occupancy probability of ocelots in RDSP (Table 3). The ocelot detection probability showed a strong and positive association with the dry season (*w_+_ *= 0.87; Figure 2a; Table 3) and native grasslands (*w_+_* = 0.86; Figure 2b; Table 3). None of the other covariates influenced the detection probability of ocelots (*w*_+_ < 0.50; Table 3).

**TABLE 3:** Cumulative weights of AICc (*w_+_*) in decreasing order for each covariate used to model the probabilities of occupancy (Ψ) and detection (*p*) of ocelots in the Rio Doce State Park, State of Minas Gerais, southeastern Brazil

Covariates	Cumulative weights	*β* parameters			
	AICc (*w_+_*)	Estimate	*SE*	LCI (95%)	UCI (95%)
Occupancy (Ψ)					
Distance to pasture	0.19	−0.17 × 10^–3^	0.15 × 10^–3^	−0.47 × 10^–3^	0.12 × 10^–3^
Season^a^	0.14	0.07	0.80	−1.50	1.65
Distance to lake	0.12	0.14 × 10^–3^	0.27 × 10^–3^	−0.38 × 10^–3^	0.66 × 10^–3^
Native grassland	0.12	−0.04	0.07	−0.19	0.10
Distance to cities	0.11	−0.1 × 10^–4^	0.1 × 10^–3^	−0.21 × 10^–3^	0.19 × 10^–3^
Distance to Eucalyptus plantations	0.11	−0.22 × 10^–4^	0.16 × 10^–3^	−0.34 × 10^–3^	0.3 × 10^–3^
Distance to river	0.11	0.24 × 10^–4^	0.67 × 10^–4^	−0.11 × 10^–3^	0.15 × 10^–3^
Detection (*p*)					
Season^a^	**0.87**	−0.98	0.34	−1.64	−0.32
Native grassland	**0.86**	0.13	0.04	0.06	0.20
Distance to lake	0.20	−0.13 × 10^–3^	0.15 × 10^–3^	−0.44 × 10^–3^	0.17 × 10^–3^
Distance to Eucalyptus plantations	0.19	0.86 × 10^–4^	0.11 × 10^–3^	−0.12 × 10^–3^	0.29 × 10^–3^
Distance to river	0.17	0.36 × 10^–4^	0.37 × 10^–4^	−0.37 × 10^–4^	0.11 × 10^–3^
Mean rainfall	0.15	0.03	0.03	−0.03	0.08
Distance to cities	0.13	0.61 × 10^–6^	0.67 × 10^–4^	−0.13 × 10^–3^	0.13 × 10^–3^
Distance to pasture	0.13	−0.47 × 10^–4^	0.81 × 10^–4^	−0.21 × 10^–3^	0.11 × 10^–3^

Note

The estimates of the *β* parameters (i.e., effects of the covariates) were extracted from the most parsimonious model containing the covariate. The weights of AICc in bold represent a strong evidence of the response of the ocelots to the covariate (*w*_+_ ≥ 0.50). *SE* = standard error; LCI = 95% lower confidence interval; UCI = 95% upper confidence interval. Distance to pasture = distance between the sampling station and the nearest pasture; season = season (dry or rainy) sampled; distance to lake = distance between the sampling station and the nearest lake; native grassland = native grassland areas within a 500‐m‐radius buffer around each sampling station; distance to cities = distance between the sampling station and the nearest city; distance to Eucalyptus plantations = distance between the sampling station and the nearest Eucalyptus plantation; distance to river = distance between the sampling station and the nearest river; mean rainfall = mean rainfall in each sampling occasion at each station.

^a^ Beta parameter value based on the rainy season.

## 4 DISCUSSION

Our results give support to a strong and positive influence of the dry season on ocelot detection in RDSP. We believe that other factors related to resources availability between seasons could be increasing ocelot detection in the dry season. These factors could be related, for example, to a higher number of individuals in the Park during this season. In fact, a study conducted in the Park during the same period reported a higher ocelot density in the dry season (Arrais, 2019), and more individuals moving through the Park could have increased ocelot detection probability during this season. Another possibility is that some prey species might change their availability between seasons due to factors that are not necessarily related to the mean rainfall, but that could also be influencing ocelot detection between seasons.

The detection of ocelots showed a strong and positive association with native grasslands, indicating that ocelots use these areas more intensively. This is contrary to our hypothesis of less detectability in native grassland areas as ocelots usually do not use grassland areas and are normally associated with forested areas (Cruz et al., 2019; Massara et al., 2015; Paolino et al., 2018). However, ocelots have been reported to use forest edges to hunt (Davis et al., 2011) and have been recorded hunting in open areas at night (Sunquist & Sunquist, 2002). Thus, it is possible that ocelots in RDSP use grassland areas for opportunistic hunting of prey more frequently than forested areas. Another possibility is that ocelots may use these areas as travel routes because most grassland areas in RDSP are around lakes, and thus may be more detected by camera traps (Figure 2).

Contrary to our prediction, none of the covariates studied influenced ocelot occupancy probability. The RDSP has little heterogeneity of natural environments, being composed mainly of high‐quality forest and water bodies. This can be observed through the presence of continuous forest that covers the entire Park and several permanent water bodies (IEF, 2019) (Figure 1). The continuous high‐quality forest and high availability of water (lakes and rivers) within RDSP may have minimized the potential effects of cities, pasture, Eucalyptus plantations and water bodies on ocelot occupancy and detection probabilities. Specifically, for the rainfall covariate, levels of precipitation in the region of RDSP during our study were much below the expected average for the rainy season (INMET, 2018). Thus, the shortage of precipitation during the rainy season may have minimized the potential effects of rainfall on ocelot detection probability. Therefore, we believe that the high‐quality and homogeneity of natural resources throughout the Park may have influenced the lack of effect of our covariates on ocelot occupancy and detection probabilities.

The ocelot occupancy estimate found in our study was smaller than those occupancy estimates found in other protected forested areas, including the estimate from another research conducted in RDSP (see Carvalho et al., 2019; Massara et al., 2018; Santos et al., 2019; da Silva et al., 2018). Because our sampling design covered the entire RDSP area, we believe that our ocelot occupancy estimate reflected the real occupancy estimate for the species in the Park. When compared to estimates found in human‐altered environments or nonprotected areas (Cruz et al., 2018; Lombardi et al., 2020), our occupancy estimate was considerably higher. It is worth noting that RDSP is one of the largest Atlantic Forest remnants in Brazil; hence, the ocelot occupancy estimate found in this study might reflect a positive scenario for the species in the biome. Thus, we believe the scenario for the ocelot in the Atlantic Forest might be alarming, once the majority of the Atlantic Forest fragments are much smaller than RDSP and/or are not under any level of protection (Ribeiro et al., 2009). Our results also indicate that ocelots do not occupy the entire Park, suggesting that some factors that were not considered in this study might be limiting its presence throughout the Park. Since the entire Park is homogeneously covered by forest, it is unlikely that vegetation structure could be affecting ocelot occupancy. Prey availability and/or coexistence with larger predators could have been affecting ocelot occupancy, as reported in other studies (Massara et al., 2018; Santos et al., 2019). However, we did not evaluate the effects of these covariates in the present study. Our sampling design was focused on detecting medium and large‐sized mammals, thus biasing the detection of ocelots' main prey species (small mammals—Bianchi et al., 2012). As for large predators, we recorded the puma in our sampling stations. However, we only had a few records of the species by sampling station (0–4 registers), which prevented us from evaluating any possible large predator effect on ocelot occupancy.

In general, no covariate affected the occupancy of ocelots, while only season and native grassland areas were responsible for affecting the detection of ocelots in RDSP. Considering the fact that the RDSP is a highly forested and relatively large strictly protected area and one of the largest and most preserved Atlantic Forest fragments in Brazil, containing a high biological diversity (Keesen et al., 2016; Silva Júnior et al., 2010; Stallings et al., 1991), our study indicates that the RDSP still provides a structurally suitable forest habitat for ocelots. However, because of the current worrying scenario of over fragmentation, reduction of forest cover, and weakness of the protective legislation of this biome, the long‐term persistence of the species in RDSP is uncertain. Also, future studies in RDSP should sampled during a wider period, encompassing more temporal replicates of the dry and wet seasons, and use different methodologies (e.g., diet analysis and a specific small mammal sampling protocol) to investigate how ocelot main prey are distributed in the Park and whether their distributions may vary between seasons. It may help differentiate among the hypothesized mechanisms of our findings of increased ocelot detection probability (i.e., here interpreted as intensity of use) in the dry season and also in native grassland areas.

## CONFLICT OF INTEREST

None declared.

## AUTHOR CONTRIBUTIONS

**Juliana Benck Pasa:** Conceptualization (supporting); formal analysis (lead); methodology (supporting); writing–original draft (lead); writing–review and editing (supporting). **Ricardo Corassa Arrais:** Investigation (equal); investigation (equal); methodology (supporting). **Rodrigo Lima Massara:** Formal analysis (supporting); methodology (supporting); writing–review and editing (supporting). **Gabriel Pereira:** Methodology (supporting); resources (supporting); writing–original draft (supporting); writing–review and editing (supporting). **Fernando Cesar Cascelli de Azevedo:** Conceptualization (lead); funding acquisition (lead); investigation (equal); methodology (equal); project administration (lead); resources (lead); supervision (lead); writing–original draft (supporting); writing–review and editing (lead).

## Data Availability

The data that support the findings of this study are available as Supplementary Information.

## 1

**TABLE A1:** Pearson's correlation test between the pre‐selected covariates for modeling the ocelot occupancy and detection probabilities in Rio Doce State Park, state of Minas Gerais, southeastern Brazil

	River	Lake	City	Eucalyptus	Pasture	Grassland	Mean_rain
River	–	−0.15	−0.25	0.04	−0.24	0.07	0.42
Lake		–	−0.28	−0.20	−0.40	−0.38	−0.47
City			–	0.26	0.52	0.33	0.33
Eucalyptus				–	0.13	0.34	0.09
Pasture					–	0.17	0.33
Grassland						–	0.34
Mean_rainfall							–

Note

Covariates highly correlated (*r* > 0.6) were removed from the analysis (indicated below with an asterisk). River = distance between the sampling station and the nearest river (in meters); Lake = distance between the sampling station and the nearest lake (in meters); City = distance between the sampling station and the nearest city (in meters); Eucalyptus = distance between the sampling station and the nearest Eucalyptus plantation (in meters); Pasture = distance between the sampling station and the nearest pasture (in meters); Grassland = native grassland areas within a 500‐m‐radius buffer around each sampling station; and Mean_rain = mean rainfall.
